# Air Pollution by Hydrothermal Volcanism and Human Pulmonary Function

**DOI:** 10.1155/2015/326794

**Published:** 2015-08-02

**Authors:** Diana Linhares, Patrícia Ventura Garcia, Fátima Viveiros, Teresa Ferreira, Armindo dos Santos Rodrigues

**Affiliations:** ^1^Department of Biology, University of the Azores, Ponta Delgada, 9501-801 Azores, Portugal; ^2^CVARG, Center for Volcanology and Geological Risks Assessment (CVARG), University of the Azores, Ponta Delgada, 9501-801 Azores, Portugal; ^3^CE3C, Centre for Ecology, Evolution and Environmental Changes (CE3C) and Azorean Biodiversity Group, University of the Azores, 9501-801 Ponta Delgada, Portugal; ^4^Department of Geosciences, University of the Azores, Ponta Delgada, 9501-801 Azores, Portugal

## Abstract

The aim of this study was to assess whether chronic exposure to volcanogenic air pollution by hydrothermal soil diffuse degassing is associated with respiratory defects in humans. This study was carried in the archipelago of the Azores, an area with active volcanism located in the Atlantic Ocean where Eurasian, African, and American lithospheric plates meet. A cross-sectional study was performed on a study group of 146 individuals inhabiting an area where volcanic activity is marked by active fumarolic fields and soil degassing (hydrothermal area) and a reference group of 359 individuals inhabiting an area without these secondary manifestations of volcanism (nonhydrothermal area). Odds ratio (OR) and 95% confidence intervals (CIs) were adjusted for age, gender, fatigue, asthma, and smoking. The OR for restrictive defects and for exacerbation of obstructive defects (COPD) in the hydrothermal area was 4.4 (95% CI 1.78–10.69) and 3.2 (95% CI 1.82–5.58), respectively. Increased prevalence of restrictions and all COPD severity ranks (mild, moderate, and severe) was observed in the population from the hydrothermal area. These findings may assist health officials in advising and keeping up with these populations to prevent and minimize the risk of respiratory diseases.

## 1. Introduction

About 10% of the worldwide population inhabits or lives in the vicinity of some active or historically active volcano [1]. Despite the hazards associated with volcanic activities, the richness of soils in nutrients attracts people to live in these areas. Several studies have established an association between acute [2–4] and long-term [5, 6] exposure to anthropogenic air pollutants and lung function, while only few have analyzed the respiratory effects from volcanogenic air pollution [7–9].

**Figure 1:**
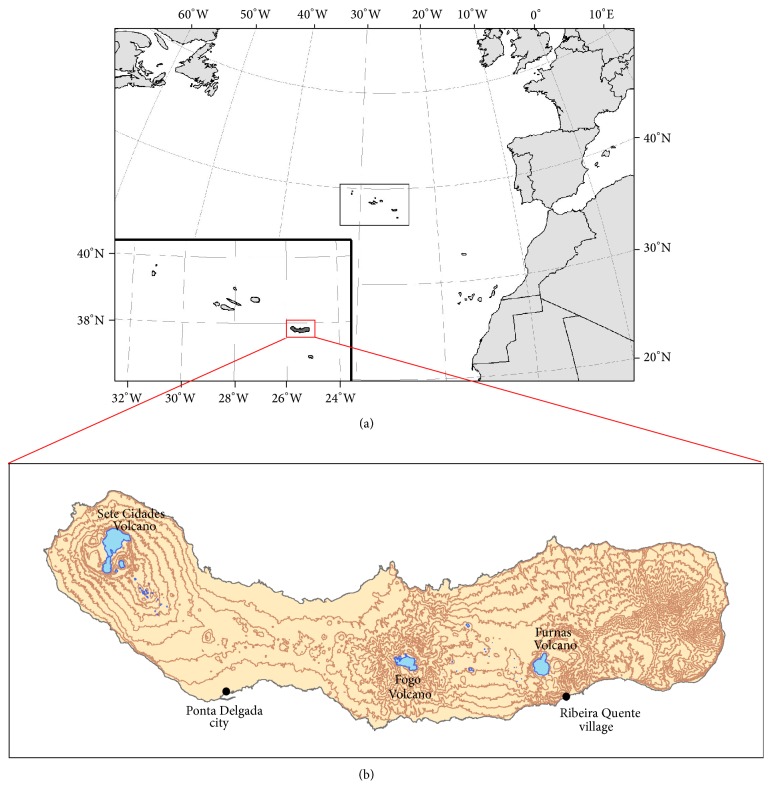
(a) Location map of the Azores archipelago and (b) São Miguel Island. The places represented on the map correspond to the two studied areas (Ponta Delgada and Ribeira Quente).

The Azores archipelago (Portugal) comprises nine volcanic inhabited islands, located between 36°45′–39°45′N and 24°45′–31°17′W (Figure 1(a)), where the Eurasian, African, and American lithospheric plates meet [10]. On account of this complex tectonic setting, seismic and volcanic activities are frequent in the archipelago [11]. São Miguel Island, the largest of the archipelago, is formed by three major active central volcanoes (Sete Cidades, Fogo, and Furnas), linked by rift zones [12] (Figure 1(b)). Furnas Volcano is located in the eastern part of the island, where present-day volcanic activity is marked by several hydrothermal manifestations consisting of active fumarolic fields, thermal and cold CO_2_-rich springs, and soil diffuse degassing areas [11, 13]. Gases released in these diffuse degassing areas are essentially carbon dioxide (CO_2_) and radon (^222^Rn), this last one a radioactive gas. Carbon dioxide is one of the most abundant volcanic gases and is amongst the most important diffused gases released by soil degassing in Furnas Volcano (hydrothermal soil CO_2_ emissions in Furnas Volcano are estimated to be approximately 968 t/d) [14]; this gas, if present at high concentrations, can become particularly dangerous for public health, since it works as asphyxiant preventing oxygen respiration [15]. Previous studies [13] showed that CO_2_ is released permanently to the atmosphere from soils in volcanic areas not only during eruptive periods, but also during quiescent periods of activity. Considering that CO_2_ released by soils may enter the buildings through pipes, cracks in the floor, and/or the contact between floor and walls, it is considered important to assess the CO_2_ flux in buildings. Carbon dioxide level is usually greater inside a building than outside, and it can act as an indicator of ventilation efficiency, showing whether the supply of outside air is sufficient to dilute indoor air contaminants [16]. According to WHO [17], indoor air pollution is responsible for 2.7% of the diseases worldwide; such effects of indoor air pollution are particularly highlighted in studies regarding the occupational exposure, as it was shown in the review made by Balmes et al. [18] that estimated that 15% of COPD was attributable to the air quality at the workplace.

Furnas and Ribeira Quente are two villages located, respectively, inside the caldera and in the south flank of Furnas active volcano, where the ground gas emissions that characterize the diffuse degassing areas occur permanently and, thus, inhabitants of such areas are often exposed to elevated concentrations of CO_2_ from volcanic origin [14]. Previous studies evidenced that Furnas inhabitants have a high incidence of chronic bronchitis and of some cancer types (e.g., lip, oral cavity, and pharynx) [19, 20] and a higher risk of DNA damage in human buccal epithelial cells [21]. Moreover, a very recent study by Camarinho et al. [22] showed that chronic exposure to volcanogenic air pollutants causes lung injury in wild mice. However, to our knowledge, up to date no study was carried out to assess the association between volcanogenic air pollution by soil diffuse degassing (DDS) and the risk of development of respiratory defects. Therefore, the present study was carried out to evaluate whether chronic exposure to permanent volcanogenic air pollution is a risk factor for human restrictive and obstructive (COPD) respiratory defects.

## 2. Methods

### 2.1. Study Population

To perform this study, two areas were selected: an area with no secondary manifestations of volcanism, therefore a nonhydrothermal area (Ponta Delgada), and an area where volcanic activity is marked by active fumarolic fields and degassing soils (hydrothermal area) (Ribeira Quente). A diagnosis campaign was established for evaluation of pulmonary function by spirometry tests for the inhabitants of both areas. Spirometry tests were carried out in either the participants' workplaces or their homes. A standard questionnaire was applied to each individual that volunteered to participate. Medical history data for respiratory symptoms were taken using a standard questionnaire modified from a standardized respiratory symptom questionnaire from American Thoracic Society (ATS) [23] and British Medical Research Council's Committee [24]. Each person was interviewed about their age, height, weight, education, occupation, smoking habits (smoking of cigarettes and/or use of smokeless tobacco), amount of cigarettes smoked/day, fatigue, and general respiratory health status, where asthma was defined by a positive response to “Did any doctor diagnose you with asthma?”.

The study group consisted of 146 participants (94 women and 52 men), residents in Ribeira Quente village (Table 1). Ribeira Quente village has 767 inhabitants [25]: 148 children and 619 adults (298 women and 321 men); therefore, the participation rate was 26.3%. This village is located on the south flank of Furnas Volcano (São Miguel Island) (Figure 1(b)), in an important diffuse degassing structure (DDS); about 98% of buildings in this village are placed above anomalous soil CO_2_ degassing with volcanic-hydrothermal origin [14, 26]. In addition, several fumaroles may be found along Ribeira Quente stream and dispersed in the village [13]. Thus, Ribeira Quente inhabitants are chronically exposed to gases of volcanic origin, particularly from soil diffuse degassing. Even if no indoor measurements were performed concomitantly to the diagnosis tests, previous works showed that in the diffuse degassing structures indoor CO_2_ is anomalously high and can reach lethal values during extreme weather events [13]. Permanent release of gases from the soils increases the indoor CO_2_ concentrations independently of the anthropogenic contribution.

The reference group comprised 359 individuals (204 women and 155 men) working in downtown of Ponta Delgada city (Table 1). Ponta Delgada's downtown has 7818 adult workers [25]; therefore, the participation rate was 4.5%. Ponta Delgada is located in the south side of São Miguel Island (Azores, Portugal; Figure 1(b)), in the Região dos Picos Volcanic Complex, a basaltic rift zone located between Sete Cidades and Fogo Volcanoes, where no manifestations of volcanism have been identified [11].

The Ethics Board of Divino Espirito Santo Hospital (Ponta Delgada, Azores, Portugal) approved the study. All individuals signed a written informed consent, in compliance with the Helsinki Declaration and Oviedo Convention, to participate in this study. Table 1 summarizes the demographic characteristics and main lifestyle habits of the studied populations. Only individuals resident for more than five years in each locality were considered in this study.

### 2.2. Spirometry Tests

The forced expiratory volume in one second (FEV_1_) and the forced vital capacity (FVC) values were obtained by spirometry in all participants. Spirometry tests were conducted with the participants in an up position wearing a nose clip and a disposable mouth piece using the EasyOne automated portable spirometer (ndd, Zürich, Switzerland), which meets ATS/ERS spirometry standards [27] and is equipped with software that checks for unacceptable maneuvers and compares the measured values with reference tables. Standardized operating procedures were implemented and controlled. Participants performed three to five attempts to provide at least three technically acceptable maneuvers, following the criteria recommended by the ATS [28] and the guidelines of the European Respiratory Society [27]. Postbronchodilator tests were not applied.

Spirometry data were classified categorically as being consistent with either a normal pulmonary function, a restrictive defect, or an obstructive defect. Spirometric detection of restriction was considered in all subjects with normal FEV_1_/FVC, with FVC < 80% predicted, and FEV_1_/FVC < 70% was used as a fixed cut-point for obstruction (COPD), according to the third United States National Health and Nutrition Examination Survey (NHANES III) for adult Caucasians. According to the GOLD guidelines, COPD was further classified in the following ranks given by the spirometer output: mild (FEV_1_ ≥ 80%), moderate (FEV_1_ 50–79%), and severe (FEV_1_ 30–49%).

### 2.3. Exposure Assessment to Volcanogenic Air Pollution by DDS

Carbon dioxide degassing maps may be useful for volcanic/seismic monitoring purposes as they represent a reference for future variations on the state of activity of the volcano [14].

Measurements of soil CO_2_ released by soil degassing in Ribeira Quente village were carried out recently by Viveiros et al. [14, 26]. The surveys were made using portable instruments that perform measurements based on the accumulation chamber method [29]. Almost all buildings (about 98%) in Ribeira Quente village are placed in an anomalous high CO_2_ degassing zone (DDS), with soil CO_2_ fluxes that can reach values higher than 25000 g/m^2^/d [14]. Considering that CO_2_ released by soils may enter the buildings (e.g., through pipes, cracks in the floor, and/or the contact between floor and walls), CO_2_ degassing maps produced for Ribeira Quente village [14, 26] were used to attribute a CO_2_ flux value to each building. Even if no indoor measurements were performed, the CO_2_ released from soils is positively correlated with the indoor CO_2_ concentrations [13], so measurements applied outside the buildings are indirectly representative of the CO_2_ concentrations that can be found indoors [13]. Sporadic soil CO_2_ flux measurements were performed at Ponta Delgada in the areas surrounding the buildings occupied by individuals from the reference group. The 68 measurements performed showed that CO_2_ flux values were lower than 25 g/m^2^/d and thus representative of biogenic origin and without hydrothermal contribution.

### 2.4. Statistical Analysis

Pearson Chi-Square test was used to compare restrictions and COPD prevalence between individuals inhabiting the environment with volcanic degassing and individuals from the reference group. To estimate the association between chronic exposure to an environment with volcanic air pollution and restrictive and obstructive defects (no versus yes), odds ratio (OR) and 95% confidence intervals (95% CIs) were calculated using a binary logistic regression model, adjusting for age, gender (male versus female), fatigue (yes versus no), and smoking status (yes versus no).

To estimate the association between chronic exposure to an environment with volcanic air pollution and the increase in the severity COPD, odds ratio (OR) and 95% confidence intervals (95% CIs) were calculated using an ordinal logistic regression model, adjusting for age, fatigue (yes versus no), asthma (yes versus no), and smoking status (yes versus no). The occurrence of obstructions was graded on scales, according to their occurrence and severity: 1: without obstruction, and 2 to 4: with obstruction, from the least severe (2) to the most severe (4).

Mann-Whitney *U* test was used to compare soil CO_2_ fluxes released by diffuse degassing between the reference and the study group.

All statistical analysis was performed using IBM SPSS Statistics 20.0 for Windows [30], and the level of statistical significance was set at *P* ≤ 0.05.

## 3. Results

The general characteristics of the study populations are presented in Table 1. The study group has an older population and a higher BMI than the reference one (56.1% versus 42.3% of individuals with more than 41 years and 27.1 kg/m^2^ versus 25.7 kg/m^2^, resp.). On the other hand, the reference group has a higher percentage of smokers than the study group (37.6% versus 19.1%).

Soil CO_2_ flux was significantly different (*P* < 0.001) between the studied areas (Table 1). According to criteria for diffuse degassing susceptibility areas defined by Viveiros et al. [14], all the analyzed buildings in Ponta Delgada are located in low susceptibility areas (CO_2_ flux < 25 g/m^2^/d), while in Ribeira Quente 1.9% of the buildings were located in moderate susceptibility areas (soil CO_2_ flux between 25 g/m^2^/d and 50 g/m^2^/d) and the remaining 98.1% were in a high susceptibility area (soil CO_2_ flux ≥ 50 g/m^2^/d) (Supplementary Material 1 in Supplementary Material available online at http://dx.doi.org/10.1155/2015/326794).

### 3.1. Prevalence of Restrictive and Obstructive Respiratory Defects

**Figure 2:**
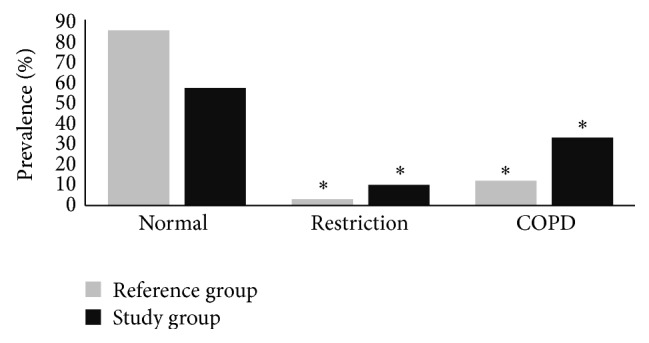
Proportion (%) of individuals with obstructive and restrictive airway diseases in study (Ribeira Quente) and reference (Ponta Delgada) groups;  ^*^significantly different at *P* < 0.05.

The prevalence of restrictions in the study group was significantly higher than in the reference group (10.2% versus 3.0%, resp.; *P* = 0.001). Similarly, the prevalence of COPD was significantly higher in the study group than in the reference one (33.6% versus 11.9%, resp.; *P* < 0.001) (Figure 2). The prevalence of more severe obstructions was also higher in the study group compared to the reference one (mild, 15.7 versus 4.4, moderate, 6.8 versus 2.2, and severe, 4.7 versus 0, resp.).

### 3.2. Restrictive or Obstructive Respiratory Defects and Exposure to Volcanogenic Air Pollution by DDS

Exposure to volcanogenic air pollution by DDS was a significant predictor of the prevalence of respiratory restrictions and of COPD exacerbation in the multivariate analysis. After adjustment for age, gender, fatigue, and smoking status, a higher prevalence of respiratory restrictions was found associated with exposure to volcanogenic air pollution by DDS (OR = 4.4; 95% CI, 1.78–10.69; *P* = 0.001) (Table 2). Also, after the adjustment for the same factors, a higher prevalence of respiratory obstructions was found associated with exposure to the volcanic environment (OR = 2.8; 95% CI, 1.60–4.99) (Table 3). Exacerbation in COPD severity was also found significantly associated with exposure to volcanogenic air pollution by DDS (OR = 3.2; 95% CI, 1.82–5.58; *P* < 0.001) (Table 4).

The analyzed confounding factors did not show any significant association with respiratory restrictions (Table 2), but respiratory obstructions were significantly associated with age, fatigue, and smoking status (Table 3). The increase in COPD severity was significantly associated with asthma and the abovementioned factors (Table 4).

## 4. Discussion

The association between exposure to air pollution and adverse respiratory effects has been widely demonstrated [31, 32]. Such association is usually related to anthropogenic air pollution, while for volcanogenic pollution there are much fewer studies. Volcanoes and volcanic manifestations, such as fumaroles and hot and cold CO_2_-rich springs as well as degassing soils, release into the environment metals, hazardous aerosols, and gases that daily affect the quality of the environment and the health of human populations [33].

The measurements of CO_2_ flux revealed that the study group (from the hydrothermal area) is chronically exposed to elevated volcanogenic air pollution by soil diffuse degassing compared to the reference group (average values are 508 g/m^2^/d (≈35.73 ppm/s) versus 15.36 g/m^2^/d (≈1.08 ppm/s), resp.). Carbon dioxide levels are described to be usually greater inside a building than outside [34, 35]; therefore, these populations are much probably subjected to higher levels of indoor CO_2_ than the presented values. Also, independently of the biological CO_2_ contribution, the hydrothermal CO_2_ emission is permanent and thus the level of CO_2_ in the Ribeira Quente buildings must be always higher than the “normal” values and sporadically reach anomalous high values as recognized in previous works, only due to meteorological changes [13]. The threshold limit value for 8 h time weighted-average exposures to CO_2_ is 5000 ppm (WHO), and possibly the average CO_2_ concentrations encountered in the buildings are below this limit and are not expected to cause health symptoms such as headaches, fatigue, and eye and throat irritation. However, the consequences of the permanent presence of indoor CO_2_ in concentrations higher than the outdoor environment are still far from being understood and may eventually lead to some stress effect in the organisms that needs to be better studied. In addition, the CO_2_ emission may be also associated with other volcanic emissions, as it is the case of the radioactive gas radon. The measurements performed showed that the study group is chronically exposed to elevated volcanogenic air pollution, namely, high CO_2_ soil diffuse degassing; high concentrations of CO_2_ can cause headaches due to hypercapnia and the decrease of O_2_ in the bloodstream [36]. When these two events combine, the body responds by increasing blood flow to the brain by dilating the blood vessels, resulting in pain [36]. A significant association was found between restrictive respiratory defects and the exposure to volcanogenic air pollution by DDS, measured as 4.4 times higher in the inhabitants of Ribeira Quente (study group), when compared to the reference one. A significant association was also found between obstructive respiratory defects and exposure to volcanogenic air pollution by DDS, measured as 2.8 times higher in the study group. None of the analyzed confounding factors (age, gender, fatigue, and smoking status) was significantly associated with respiratory restriction, but apart from gender all confounding factors were positively associated with respiratory obstructions.

Results also showed significant associations between the increase in the severity of COPD and the exposure to soil diffuse degassing in the study group when compared to the reference one, given as 3.2 times higher in Ribeira Quente inhabitants (study group). Age, asthma, fatigue, and smoking status were also significantly associated with the increase in COPD severity.

Age and smoking habits are well associated with COPD prevalence [37, 38]. According to our results, the classification of subjects who are at risk is relevant in middle-aged and elderly persons (i.e., > 41 years old), individuals with asthma, and smokers. Aging affects the structure, function, and control of the respiratory system; according to Stanojevic et al. [39], both FEV_1_ and FVC decrease with age, and thus the observed increase in COPD associated with age was expected (since older individuals will have a reduced FVC and a reduced FEV_1_/FVC ratio). Also recently results from Eurostat [40] demonstrated that respiratory diseases such as bronchitis and asthma affect mainly older people, as almost 90% of EU-28 deaths from these diseases occur among those aged 65 and above. Moreover, this report presents a standardized death rate from diseases of the respiratory system of at least 150 deaths per 100 000 inhabitants; the highest death rates were observed in two Portuguese regions, the volcanic islands of Madeira (294.6 deaths per 100 000 inhabitants) and Azores (195.8 deaths per 100 000 inhabitants); the rest of the Portuguese territory (mainland) presented a death rate below 150 deaths per 100 000 inhabitants; therefore, the observed results are in accordance with this general overview.

Regarding asthma, there might be a significant overlap with COPD, since both are characterized by an underlying airway inflammation, although the structural and pathophysiologic findings in both diseases can be usually differentiated [41]. According to the Global Initiative for Chronic Obstructive Lung Diseases [42], in individuals with chronic respiratory symptoms and fixed airflow limitation, it is difficult to differentiate asthma from COPD. However, there is epidemiological evidence that long-standing asthma can lead to fixed airflow limitation [43], which increases the frequency of asthma attacks [44].

Fatigue is considered the second most common symptom of COPD, after breathlessness [45]. The increased level of fatigue found in patients with COPD is associated with an increase in the severity of lung impairment and a reduction in exercise tolerance. Symptoms of fatigue can be physical and mental, such as lack of energy or poor concentration. Baghai-Ravary et al. [46] and Breslin et al. [47] found a relationship between fatigue and COPD, which persists at COPD exacerbations. Since COPD was observed mainly in elderly people that have diminished lung function and therefore a reduction in health status, fatigue was expected to be associated with COPD.

It is well established that tobacco is the most important causative factor for COPD, with individual susceptibility continuously interacting synergistically with other risk factors [48], such as age. According to Lundbäck et al. [49], lifelong smoking increases the chance of developing COPD and, thus, about 50% of the smokers eventually develop COPD during their lifetime. However, according to GOLD [42], other risk factors of COPD include exposure to air pollution, secondhand smoke and occupational dusts and chemicals, heredity, a history of childhood respiratory infections, and socioeconomic status. In the present study, air pollution by hydrothermal volcanism was proven to be a risk factor in COPD exacerbation.

It is well known that air pollution can perpetuate a chronic inflammatory process that potentially leads to lung diseases [31]. However, the majority of studies regard air pollutants of anthropogenic origin, such as PM_10_, NO_2_, SO_2_, and O_3_ [50, 51], while only few studies were developed in areas with volcanic activity and associate volcanogenic pollutants with human airway diseases [7, 52]. Longo and Yang [52] and Iwasawa et al. [8] reported a higher risk of acute bronchitis across the lifespan in humans exposed to sulfurous volcanic air pollution. Furthermore, in 2013, Camarinho et al. [22] showed that noneruptive active volcanism is associated with increased lung injury in mice. Thus, it is not unexpected to observe an increase in the exacerbation of COPD severity in the population living in Ribeira Quente, a volcanic area with elevated soil diffuse degassing. These findings also corroborate the study by Amaral and Rodrigues [19] that observed higher rates of chronic bronchitis in Furnas village inhabitants (in comparison to a reference group) and suggested that it could be partially associated with chronic exposure, in a very humid atmosphere, to environmental factors resulting from volcanic activity.

## 5. Conclusions

To our knowledge, this is the first study assessing long-term effects of CO_2_ emissions in hydrothermal areas on the development of respiratory defects. The results of this study show that long-term exposure to air pollution by soil diffuse degassing increases the risk of developing restrictive defects as well as the exacerbation of COPD in the inhabitants of hydrothermal volcanic areas. Therefore, mitigation measures should be implemented in populations inhabiting elevated diffuse degassing areas, such as the construction of natural/forced ventilation systems, as well as follow-up health programs in order to provide medical counseling when necessary. The results of the study also support the need to particularly follow up the asthmatic and the older individuals, since these factors are associated with exacerbation in COPD severity in individuals chronically exposed to volcanogenic soil diffuse degassing.

In addition, considering the positive trend of the CO_2_ in the atmosphere, areas as Ribeira Quente village can be used as natural analogous to studying the possible effects of these increases on the population.

## Supplementary Material

Supplementary Material 1 represents the volcanogenic soil CO_2_ diffuse degassing map from Ribeira Quente village (adapted from Viveiros et al. 2010). The dots represent the 146 studied individuals and their respiratory test outcomes (normal outcome - green; restrictive defect- yellow; COPD -red).

## Figures and Tables

**Table 1:** Description of the study populations (study and reference groups) (mean ± SE for continuous variables or *n* (%) for categorical variables).

		Reference group (Ponta Delgada, *n* = 359)	Study group (Ribeira Quente, *n* = 146)	*P* value^a^
**General characteristics**				
Age (years)	41.3 ± 12.7	39.8 ± 11.2	45.1 ± 15.3	0.001
Age, >41^b^	234 (46.3)	152 (42.3)	82 (56.1)	0.005
Gender, male	207 (40.9)	155 (43.1)	52 (35.6)	0.117
BMI (kg/m^2^)	26.1 ± 4.4	25.7 ± 3.9	27.1 ± 5.4	0.006
BMI, >26^c^	213 (42.2)	141 (39.3)	72 (49.3)	0.041
Smoking status				
Smoker	163 (32.2)	135 (37.6)	28 (19.1)	<0.001
Previous smoker	91 (18)	69 (19.2)	22 (15)	0.271
Easy fatigue, yes	116 (23)	40 (11.1)	76 (52)	<0.001
Asthma, yes	43 (8.5)	29 (8)	14 (9.6)	0.581
Occupation				
White collar	135 (26.7)	122 (34)	13 (8.9)	<0.001
Blue collar	256 (50.7)	233 (65)	23 (15.8)	<0.001
Other	114 (22.6)	4 (1)	110 (75.3)	<0.001
**Study**				
FEV_1_ predicted^d^	88.5 ± 20.4	92.5 ± 16.6	78.6 ± 25.2	0.001
FVC predicted	96.6 ± 19.2	98.8 ± 15	91.1 ± 26.2	<0.001
FEV_1_/FVC predicted	91.8 ± 14.2	93.8 ± 11.7	87 ± 18.1	<0.001
Lung function				
Restriction, yes	26 (5.1)	11 (3)	15 (10.2)	0.001
COPD, yes	92 (18.2)	43 (11.9)	49 (33.6)	<0.001
CO_2_ flux, g/m^2^/d^e^		15.36	508	<0.001

^a^ *P* value comparing reference and study groups, by Mann-Whitney for continuous variables and by *χ* ^2^ for categorical variables.

^b^Cut-off defined according to the mean value (i.e., 41.3 years) of the observed age distribution in the whole population.

^c^Cut-off defined according to the mean value (i.e., 26.1 BMI) of the observed BMI distribution in the whole population.

^d^Predicted values from the National Health and Nutrition Examination Survey Cohort.

^e^CO_2_ flux is expressed in mean.

**Table 2:** Adjusted association between characteristics of study participants, exposure to volcanogenic soil diffuse degassing (DDS), and restrictive lung defects.

Binomial logistic regression			Number of obs. 505 LR chi2(5) = 10.97 Prob > chi2 = 0.05
Parameter	*n* (%)	OR (95% CI)^a^	*P* value
Age		0.99 (0.96–1.02)	0.933
Gender			
Male	207 (41)	1.16 (0.50–2.69)	0.729
Female	298 (59)	1.00	
Easy fatigue			
Yes	116 (23)	0.62 (0.23–1.67)	0.349
No	389 (77)	1.00	
Smoking status			
Smoker	163 (32.3)	0.94 (0.36–2.42)	0.892
Nonsmoker	342 (67.7)	1.00	
Exposure to DDS			
Yes (study group)	146 (28.9)	4.37 (1.78–10.69)	0.001
No (reference group)	359 (71.1)	1.00	

^a^OR, odds ratio; 95% CI, 95% confidence interval.

**Table 3:** Adjusted association between characteristics of study participants, exposure to volcanogenic soil diffuse degassing, and obstructive lung defects.

Binomial logistic regression			Number of obs. 505 LR chi2(5) = 60.80 Prob > chi2 = <0.001
Parameter	*n* (%)	OR (95% CI)^a^	*P* value
Age		1.04 (1.02–1.06)	<0.001
Easy fatigue			
Yes	116 (23)	1.80 (1.05–3.28)	0.033
No	389 (77)	1.00	
Gender			
Male	207 (41)	0.66 (0.39–1.11)	0.123
Female	298 (59)	1.00	
Smoking status			
Smoker	163 (32.3)	2.34 (1.35–4.06)	0.002
Nonsmoker	342 (67.7)	1.00	
Exposure to DDS			
Yes (study group)	146 (28.9)	2.83 (1.60–4.99)	<0.001
No (reference group)	359 (71.1)	1.00	

^a^OR, odds ratio; 95% CI, 95% confidence interval.

**Table 4:** Adjusted association between characteristics of study participants, exposure to volcanogenic soil diffuse degassing, and COPD exacerbation.

Ordinal logistic regression			Number of obs. 505 LR chi2(5) = 70.19 Prob > chi2 = <0.001
Parameter	*n* (%)	OR (95% CI)^a^	*P* value
Age		1.04 (1.02–1.06)	<0.001
Easy fatigue			
Yes	116 (23)	1.80 (1.00–3.24)	0.046
No	389 (77)	1.00	
Asthma			
Yes	43 (8.5)	2.25 (1.05–4.82)	0.037
No	462 (91.5)	1.00	
Smoking status			
Smoker	163 (32.3)	2.11 (1.25–3.56)	0.005
Nonsmoker	342 (67.7)	1.00	
Exposure to DDS			
Yes (study group)	146 (28.9)	3.19 (1.82–5.58)	<0.001
No (reference group)	359 (71.1)	1.00	

^a^OR, odds ratio; 95% CI, 95% confidence interval.
