# Supplementary material for: Air Pollution by Hydrothermal Volcanism and Human Pulmonary Function
Source: Biomed Res Int. 2015 Aug 2;2015:326794. doi: 10.1155/2015/326794 (PMC4537725; doi:10.1155/2015/326794)
Supplement: Supplementary file 1 — Supplementary Material 1 represents the volcanogenic soil CO2 diffuse degassing map from Ribeira Quente village (adapted from Viveiros et al. 2010). The dots represent the 146 studied individuals and their respiratory test outcomes (normal outcome - green; restrictive defect- yellow; COPD -red). [file 326794.f1.pdf]

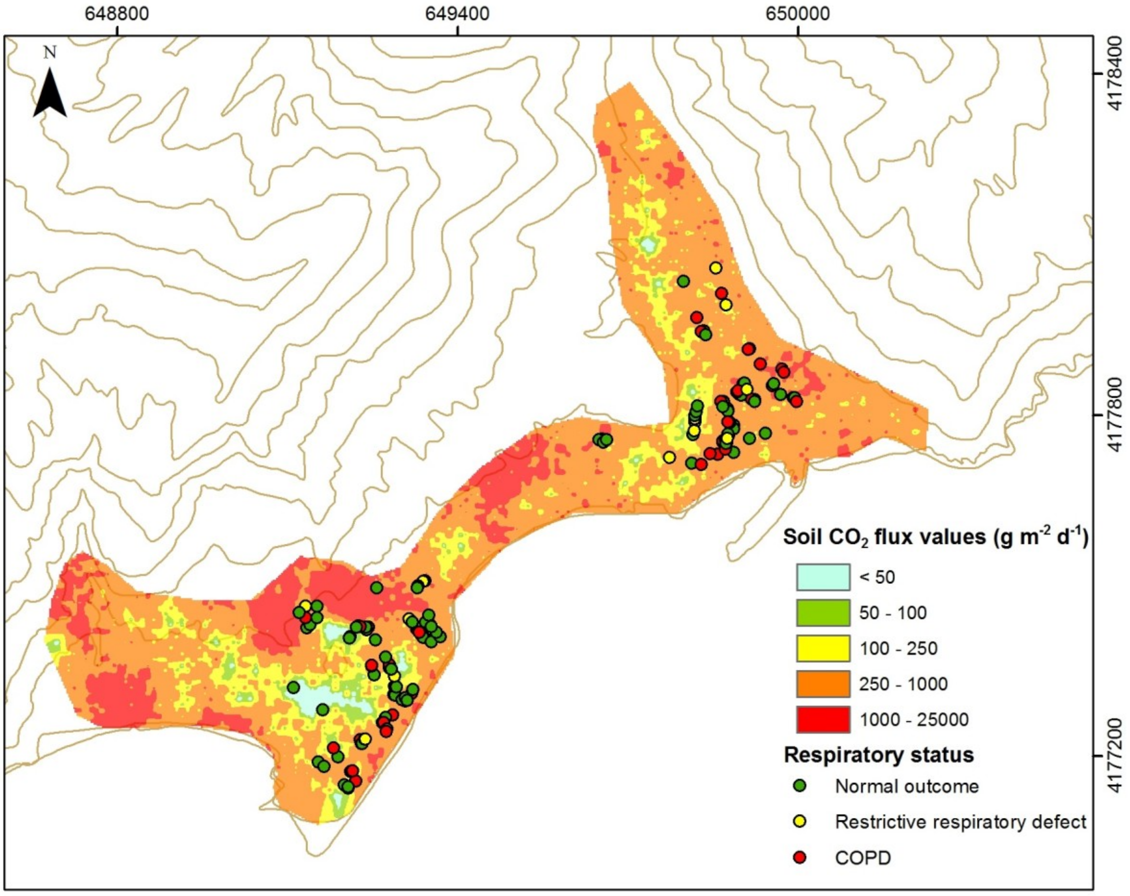

Suplemmentary material 1. Volcanogenic soil CO_2 diffuse degassing map from Ribeira Quente village (adapted from Viveiros et al. 2010). The dots represent the 150 studied individuals and their respiratory test outcomes (normal outcome - green; restrictive respiratory defect- yellow; COPD -red).
